# Pulmonary Radiological Findings in Patients with Acute Myeloid Leukemia and Their Relationship to Chemotherapy and Prognosis: A Single-Center Retrospective Study

**DOI:** 10.5152/tjh.2011.77

**Published:** 2012-09-15

**Authors:** Mehmet S Buğdacı, Halil Yanardağ, M. Cem Ar, Teoman Soysal, Süleyman Coşkun, Sabriye Demirci

**Affiliations:** 1 İstanbul University, Cerrahpaşa School of Medicine, Department of Pneumophysiology, Division of Internal Medicine, İstanbul, Turkey; 2 İstanbul Training and Research Hospital, Department of Hematology, İstanbul, Turkey; 3 İstanbul University, Cerrahpaşa School of Medicine, Department of Hematology, Division of Internal Medicine, İstanbul, Turkey

**Keywords:** Acute myeloid leukemia, Pulmonary disease, Radiological findings

## Abstract

**Objective**: Acute myeloid leukemia (AML) is the most common acute leukemia in adults. Pulmonary are among the most common causes of mortality in AML. This single-center retrospective study aimed to evaluate the relationship between radiological findings of pulmonary at presentation and post chemotherapy on prognosis and clinical outcome in a group of AML patients.

**Material and Methods:** The study included 278 AML patients. Clinical and radiological findings, laboratory findings, and microbiological culture results were evaluated. Pulmonary complications at presentation and post chemotherapy were compared.

**Results:** Pulmonary complications were observed in 53 of the patients (19%). Mean age of the patients with and without pulmonary complications was 43.1 ± 15.2 years and 38.8 ± 16.3 years, respectively (P < 0.001). Pulmonary complications were not correlated with gender, AML subtype, or the serum lactate dehydrogenase (LDH) level. The most common cause of pulmonary complications was infection. Pulmonary complications were observed in 29% and 71% of the patients at presentation and post chemotherapy, respectively.

**Conclusion:** Pulmonary complications were observed more frequently at presentation in neutropenic AML patients of advanced age. The mortality rate was higher among the AML patients that had pulmonary complications at presentation.

## Abstract (tr)

**Amaç:** Akut miyeloid lösemi (AML) erişkinde en sık görülen akut lösemidir. AML’de mortaliteyi yol açan en önemli sebeplerden biri pulmoner olaylardır. Bu retrospektif tek merkezli çalışmanın amacı tanı anında ve kemoterapi sonrası gelişen radyolojik akciğer bulgularının hastalığın klinik gidişi ve prognozu üzerine etkisinin araştırılmasıdır.

**Gereç ve Yöntemler:** Çalışmaya 278 AML hastası dahil edildi. değerlendirildi. Tanı anındaki ve kemoterapi sonrası pulmoner bulgular, hastalara ait klinik ve radyolojik bulgular, laboratuvar verileri ve mikrobiyolojik kültür sonuçları ile değerlendirildi.

**Bulgular:** Pulmoner olaylar hastaların 53’ünde (%19) görüldü. Pulmoner bulguları olan ve olmayan hastaların ortalama yaşları sırasıyla 43.1±15.2 ve 38.8±16.3 olarak bulundu (p<0,001). Akciğer bulguları ile cinsiyet, AML alt tipi ve LDH düzeyleri arasında ilişki saptanmadı. En sık rastlanılan pulmoner olay enfeksiyondu. Pulmoner olaylar hastaların %29’unda tanı anında, %71’inde tedavi sonrası gözlendi.

**Sonuç:** Pulmoner olaylar ileri yaştaki, nötropenik hastalarda tanı anında daha sık gözlendi. Tanı anında pulmoner komplikasyonu bulunan AML’li hastalarda mortalite daha yüksek bulundu.

## INTRODUCTION

Acute myeloid leukemia (AML) is a heterogeneousgroup of neoplastic disorders characterized by malignant proliferation of myeloid precursor cells. It is the most commonform of acute leukemia in adults.[1,2,3] The prognosisof acute leukemias is poor, despite improvements insupportive and definitive therapies.[4] Severe pulmonarycomplications, including acute pulmonary insufficiencyand infectious pneumonia, frequently occur in patientswith acute leukemia.[5,6] Such pulmonary complicationsmay be of infectious or non-infectious origin. Pulmonaryinfections are the most important cause of morbidity andmortality in patients with hematological malignancies undergoingchemotherapy.[7] Furthermore, high mortalityrates nearly 60% were reported in leukemia patients withradiologically evident pulmonary infiltration.[8]

The bulk of data on pulmonary findings in patientswith AML consists primarily of chemotherapy-relatedcomplications.[6,7,9,10,11] Data are lacking on other aspectsof pulmonary findings in AML, such as the associationbetween AML subtypes and pulmonary complications,and the correlation between pulmonary complications atpresentation and prognosis. As such, the aim of the presentsingle-center retrospective study was to evaluate therelationship between radiological findings of pulmonarycomplications at presentation and post chemotherapy onprognosis and clinical outcome in patients with AML.

## MATERIALS AND METHODS

Presentation and follow-up records of 1066 patients with hematological malignancies that presented to University of Istanbul, Cerrahpaşa School of Medicine, 

Hematology Department, between 1995 and 2006 were retrospectively reviewed. There were 475 patients diagnosed as acute leukemia, of which 278 with AML were included in the study. AML was diagnosed based on morphology and immunophenotyping. The patients were classified according to French-American-British (FAB) criteria. Flow cytometry was used for immunophenotyping. 

**Methodology of radiographic studies **

Thoracic high-resolution computed tomography (HRCT) was performed using a Siemens Somatom Plus (Siemens, Erlangen, Germany) scanner during breathholding at full inspiration, according to Gevenois et al [12]. At the beginning of the procedure radiographic fields were marked on digital topograms. Using a cross sectional thickness of 1 mm and a cross sectional interval of 10 mm, deep inspiration images of the whole lung parenchyma— from apex to base—were obtained using the high-spatialfrequency (bone) reconstruction algorithm. Every section over the diaphragm for each lung was evaluated separately. 

**Definitions **

Consolidation was considered homogeneous amorphous opacities observed via air bronchogram to obscure many of the underlying vessels. Ground-glass appearance was characterized as a patchy or diffuse hazy increase in lung opacity that did not obscure the underlying vessels. Radiologically, nodules are focal, rounded opacities of varying size, with either well- or ill-defined borders. In the present study, rounded opacities without any specific features were simply defined as non-specific nodular opacity. Air-containing nodules were considered cavitary nodules. Thoracic findings were classified as pleural, parenchymal, and pleuroparenchymal, according to radiological appearance. Lesions were considered parenchymal if they involved the pulmonary tissue and bronchial tree. Pleural thickening was associated with fibrosis, pleural effusion, or pleural involvement. 

Radiological, clinical, and laboratory findings were evaluated together, so as to determine their etiology. Consolidation and abscesses were considered to be due to bacterial agents initially if supportive clinical findings were present. Nodules without specific characteristics, such as a centrilobular nodule or acinar nodule (± halo finding), were considered to be of fungal origin. Cavitary lesions were diagnosed as tuberculosis if confirmed via microbiological culture results. Radiological diagnosis of pulmonary embolism was based on spiral CT findings or high-probability ventilation/perfusion scintigraphy. Invasive pulmonary aspergillosis was diagnosed according to EORTC/MSG consensus criteria.[13] Leukostasis was considered a leukocyte count >100 x 109 L–1 and the presence of pulmonary symptoms (dyspnea and diffuse interstitial infiltrates chest radiographs), only after having excluded other plausible causes. The diagnosis of bacterial pneumonia was based on the presence of a pathogen concentration >103 CFU mL–1 in culture. Respiratory findings were grouped as those that were observed at presentation and those that occurred post chemotherapy. 

**Drugs **

Cytosine arabinoside (100-200 mg m–2 on d 1-7) and daunorubicin (45 mg m–2 on d 1-3), idarubicin (12 mg m–2 on d 1-3), or mitoxantrone (12 mg m–2 on d 1-3) were administered as induction treatment for AML. High-dose cytosine arabinoside (1.5-3 g m–2 q12h on d 1, 3, and 5 [6 doses]) was administered for consolidation purposes following remission. Regimens containing all-transretinoic acid (ATRA) and idarubicin were used for remission induction and consolidation in patients with acute promyelocytic leukemia. 

Broad-spectrum beta-lactam antibiotics and carbapenems with or without aminoglycoside were administered in febrile patients with neutropenia (<500 mm–3). The addition of glycopeptide antibiotics and/or antifungal agents, as well as re-evaluation of initial antibiotic therapy, were usually carried out according to IDSA guidelines.[14,15] The study protocol was approved by the Istanbul University, Cerrahpaşa School of Medicine Ethics Committee. 

**Statistics**

Parametric variables are presented as mean ± standard deviation (mean ± SD). Non-parametric data were evaluated using the chi-square or Pearson’s correlation tests, asappropriate. Student’s t test was used for comparison ofparametric data. A P value less then 0.05 was consideredto be statistically significant. SPSS v.12.0 for Windows wasused for statistical evaluation. 

## RESULTS

The study included 278 AML patients. In all, 169 (60.7%) were male (mean age: 39.2 ± 16 years) and 109 (39.3%) were female (mean age: 40.3 ± 16.5 years). Among the patients, 241 (86.7%) had de novo AML and 37 (13.3%) had AML secondary to chronic myeloid leukemia, myelodysplastic syndrome, sideroblastic anemia, aplastic anemia, or chemotherapy. In total, 47 (16.9%) patients were aged ≥65 years. 

Pulmonary findings were observed in 53 (19.1%) of the 278 AML cases (Table 1 and the Figure). Mean age of the patients with and without radiologically confirmed pulmonary complications was 43.1 ± 15.2 years and 38.8±16.3 years, respectively; the difference was significant (P < 0.001). Pulmonary complications and gender were not statistically correlated (P = 0.96). Radiologically confirmed pulmonary complications occurred at a higher frequency in the patients with the M4 subtype (32.6%), though the difference was not significant (P = 0.55). Leukemic infiltration of the lungs was observed in 2 patients with AML M5 (n = 1) and AML M4/5 (n = 1). There wasn’t a significant difference in the serum lactate dehydrogenase (LDH) level between the patients with and without pulmonary complications (P = 0.290). 

In all, 81% of the patients had parenchymal involvement, 15.1% had pleural involvement, and 3.8% had pleuropulmonary involvement. CT findings in the patients with pulmonary lesions were as follows: ground-glass appearance (n = 18); nodular opacity (n = 15); pneumonic consolidation (n = 9); non-homogenous opacity (n = 8); fungus ball (n = 1); nodule with cavity (n = 1); abscess (n = 1). Etiological distribution of the pulmonary complications showed that in 56.6% of the cases the underlying cause was infection. Non-infectious and undetermined causes were responsible for the pulmonary complications in 24.5% and 18.8% of the cases, respectively. Clinical response to antibiotic and/or antifungal treatment was achieved in 4 of the patients with pulmonary complications of undetermined origin. 

Pulmonary complications were observed at initial presentation in 16 (28.9%) of the patients, whereas in 37 (71.1%) patients they were observed post chemotherapy. Mortality was 43.8% in the patients with pulmonary complications at presentation and 24.6% in those that developed pulmonary complications post chemotherapy. Table 2 summarizes the characteristics of the AML patients with pulmonary complications at presentation and post chemotherapy.

## DISCUSSION

Pulmonary infections are the most common cause of pulmonary radiological findings and are among the most common causes of mortality in AML.[7] Chemotherapy and allogeneic stem cell transplantation are the treatment options in patients in good general condition. The cure rate with chemotherapy alone is low and is usually not durable. Pulmonary complications are the primary cause of mortality, both before and after chemotherapy; therefore, detection of probable pulmonary involvement before commencing chemotherapy could help prevent negative outcomes that might occur during the post-treatment pancytopenic period. As such, predicting the risk of pulmonary complications in AML might play a crucial role in the success of chemotherapy by reducing complicationrelated morbidity and mortality. 

Pulmonary complications were observed in 19% of the present study’s patients, which is a lower rate than previously reported. For instance, Ewig et al.[16] observed respiratory findings in 30% of patients and Chaoui et al.[4] reported a rate of 46%. Unlike the present study, Chaoui et al.’s included patients with a higher median age (median: 55 years; range: 17-85 years) that had de novo, and secondary acute lymphoid and myeloid leukemias, which might explain why the rate of pulmonary complications was higher in their study. The most frequent cause of pulmonary complications in the present study was infections (30%), including those of bacterial and fungal origin;similar findings have been reported.[4,16,17] Ranoet al. reported that pulmonary complications were mostfrequently caused by bacterial infections, followed by fungalinfections.[18] Proven or probable invasive pulmonaryaspergillosis was observed in 95% of patients with pulmonarycomplications in the present study, whereas ratesreported in the literature are 5% in acute leukemias[19]and 6%-8.3% in AML[20].

AML is a rare cause of leukemia in individuals aged<15 years;[21] it is a disease of late adulthood and themedian age of newly diagnosed AML patients is 65 years.[22] Deschler and Lübbert reported an incidence of1.8/100,000 in patients aged <65 years and an incidenceof 17/100,000 in patients aged >65 years.[23] Mean age ofthe male and female patients in the present study was 39.2± 16 years and 40.3 ± 16.5 years, respectively.

Pulmonary leukemic infiltration was noted in 2 of thepresent study’s AML patients; 1 with the M5 subtype and1 with the M4/5 subtype. This finding in accordance withthe current knowledge. Autopsy studies reported that pulmonaryinfiltration occurs at a rate of 24%-64% in AMLM5 cases, versus 5% for clinically and radiologically diagnosedleukemic lung infiltration.[24,25] No correlationwas observed between the serum LDH level and pulmonarycomplications in the present study, which is agreementwith Chaoui et al.,[4] who reported that there wasn’ta difference in LDH levels between patients with and withoutpulmonary complications. 

In the present pulmonary complications occurred more frequently in the patients with advanced age particularly > 65-year-old and there was a statistically significant correlation between age and pulmonary complications (P = 0.001), which is similar to the findings reported by Rossini et al.[6] Unexplained causes of pulmonary complications were observed in 18.8% of the present study’s patients. Similar findings were reported by Chaoui et al. and Rano et al., who reported that pulmonary complications of unexplained origin occurred in 25% and 19% of immunocompromised patients, respectively.[4,18] Pulmonary complications of unidentified origin could be associated with the absence of sufficient diagnostic work-up due to poor general status or severe cytopenias. 

In the present study 33% of the non-infectious causes of pulmonary complications were cardiac pathologies. Cardiac failure-related pulmonary complications occurred primarily during the first week of induction chemotherapy, suggesting that the pulmonary complications were related to chemotherapy or hydration. Cardiovascular dysfunction was reported to occur easily in anemic patients receiving high-volume fluid replacement, and combination chemotherapy consisting of anthracycline and cytosine arabinoside.[26] 

The present study’s most important findings are as follows: 

1. The frequency of pulmonary complications did not differ significantly between AML subtypes; 

2. Pulmonary complications at presentation occurred more often in patients of advanced aged with a low absolute neutrophil leukocyte count. Consequently, the baseline absolute neutrophil leukocyte count is an important prognostic factor in patients with advanced age; 

3. Ground-glass appearance was the most common nonspecific radiological finding and should be considered as infection in AML patients until proven otherwise; 

4. The serum LDH level was observed to be a prognostic factor in the AML patients; however, it is a non-specific marker of pulmonary complications. 

In conclusion, the most common pulmonary complication in the present study was infection. We think that the presence of pulmonary complications at diagnosis is a predictor of poor prognosis in AML patients. 

**Conflict of Interest Statement**

The authors of this paper have no conflicts of interest, including specific financial interests, relationships, and/ or affiliations relevant to the subject matter or materials included.

## Figures and Tables

**Table 1:**
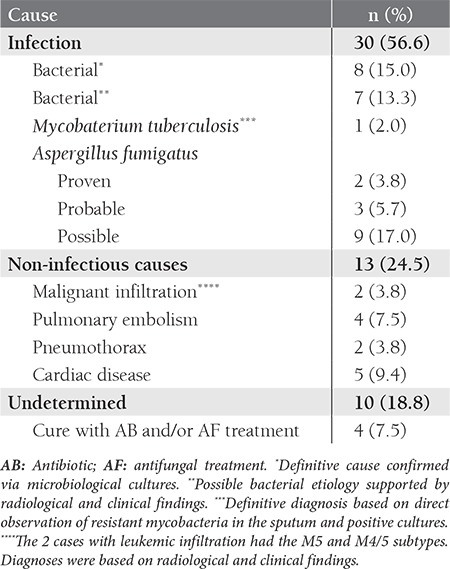
The etiology of radiologically confirmed pulmonary complications and the frequency of *Aspergillus* infection in the AML patients (n = 53), according to EORTC/MSG consensus criteria.

**Table 2:**
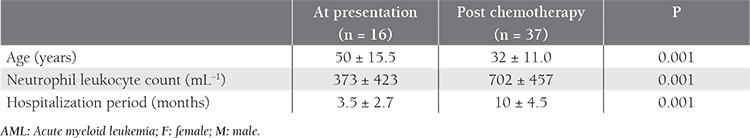
Characteristics of the AML patients with pulmonary complications at presentation and post chemotherapy.

**Figure:**
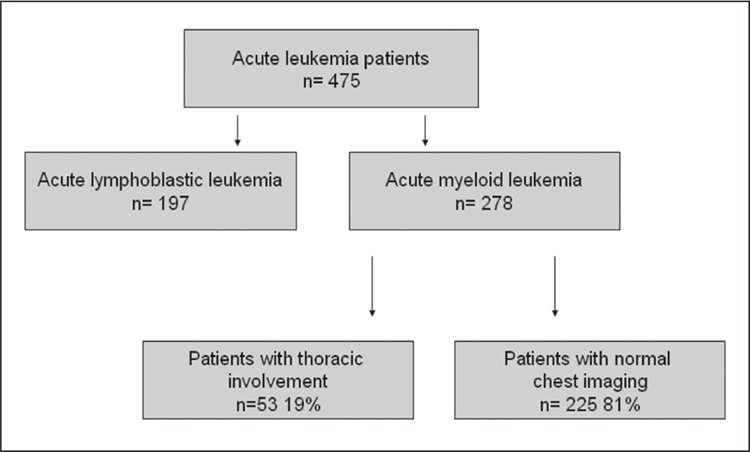
Leukemia cases included in the study and the distribu- tion of AML patients with pulmonary complications.
